# UL36 Encoded by Marek’s Disease Virus Exhibits Linkage-Specific Deubiquitinase Activity

**DOI:** 10.3390/ijms21051783

**Published:** 2020-03-05

**Authors:** Junyan Lin, Yongxing Ai, Hongda Zhou, Yan Lv, Menghan Wang, Jiacui Xu, Cong Yu, Huanmin Zhang, Mengyun Wang

**Affiliations:** 1 College of Animal Science, Jilin University, 5333 Xi An Road, Changchun 130062, Jilin, China; linjunyan919@163.com (J.L.); aiyx@jlu.edu.cn (Y.A.); hongdazhou521@163.com (H.Z.); lvyanty@jlu.edu.cn (Y.L.); menghanwang927@163.com (M.W.); jcxu@jlu.edu.cn (J.X.); 2 Key Laboratory of Zoonosis Research, Ministry of Education, College of Veterinary Medicine, Institute of Zoonosis, Jilin University, 5333 Xi An Road, Changchun 130062, Jilin, China; 3 State Key Laboratory of Electroanalytical Chemistry, Changchun Institute of Applied Chemistry, Chinese Academy of Sciences, 5625 Renmin Avenue, Changchun 130022, Jilin, China; congyu@ciac.ac.cn; 4 Avian Disease and Oncology Laboratory, Agriculture Research Service, United States Department of Agriculture, 4279 East Mount Hope Road East Lansing, MI 48823, USA

**Keywords:** UL36, deubiquitinase, MDV, catalytic activity, substrate specificity

## Abstract

(1) Background: Deubiquitinase (DUB) regulates various important cellular processes via reversing the protein ubiquitination. The N-terminal fragment of a giant tegument protein, UL36, encoded by the Marek’s disease (MD) virus (MDV), encompasses a putative DUB (UL36-DUB) and shares no homology with any known DUBs. The N-terminus 75 kDa fragment of UL36 exists in MD T lymphoma cells at a high level and participates in MDV pathogenicity. (2) Methods: To characterize deubiquitinating activity and substrate specificity of UL36-DUB, the UL36 N-terminal fragments, UL36(323), UL36(480), and mutants were prepared using the Bac-to-Bac system. The deubiquitinating activity and substrate specificity of these recombinant UL36-DUBs were analyzed using various ubiquitin (Ub) or ubiquitin-like (UbL) substrates and activity-based deubiquitinating enzyme probes. (3) Results: The results indicated that wild type UL36-DUBs show a different hydrolysis ability against varied types of ubiquitin chains. These wild type UL36-DUBs presented the highest activity to K11, K48, and K63 linkage Ub chains, weak activity to K6, K29, and K33 Ub chains, and no activity to K27 linkage Ub chain. UL36 has higher cleavage efficiency for K48 and K63 poly-ubiquitin than linear ubiquitin chain (M1-Ub4), but no activity on various ubiquitin-like modifiers. The mutation of C98 and H234 residues eliminated the deubiquitinating activity of UL36-DUB. D232A mutation impacted, but did not eliminated UL36(480) activity. The Ub-Br probe can bind to wild type UL36-DUB and mutants UL36(480)^H234A^ and UL36(480)^D232A^, but not C98 mutants. These in vitro results suggested that the C98 and H234 are essential catalytic residues of UL36-DUB. UL36-DUB exhibited a strict substrate specificity. Inhibition assay revealed that UL36-DUB exhibits resistance to the Roche protease inhibitor cocktail and serine protease inhibitor, but not to the Solarbio protease inhibitor cocktail. (4) Conclusions: UL36-DUB exhibited a strict substrate preference, and the protocol developed in the current study for obtaining active UL36-DUB protein should promote the high-throughput screening of UL36 inhibitors and the study on the function of MDV-encoded UL36.

## 1. Introduction

Ubiquitination is one of the most important regulatory machinery of post-translational modification of intracellular proteins [1]. The modifier, ubiquitin (Ub) protein, is a highly conserved small protein consisting of 76 amino acids throughout eukaryotes. One Ub could be modified by another Ub on K6, K11, K27, K29, K33, K48, K63, and M1 residues to form various types of Ub chains. The modifications by different type or length of these linkage Ub chains can confer different functions or fate to the modified protein, such as leading the modified protein to degradation, modulating signaling pathways, and regulating enzyme activity [2,3,4,5]. Besides Ub, eukaryotes also encode many ubiquitin-like (UbL) modifiers, such as SUMO-1,-2 and -3, NEDD8, FAT10, ISG15, UFM1, Hub1, etc., which share a higher structure similarity to Ub but have distinctive amino acid sequence and perform different functions from Ub [6]. Ub or UbL modifications are reversible through isopeptide bond proteases, deconjugating these modifiers from target proteins. The enzymes deconjugating UbL modifiers rarely share cross-reactivity with deubiquitinase (DUB). The rigor of these modifications is conducive to the meticulous regulation on the function of cellular proteins and increases the complexity of intracellular regulation. Thus, revealing the substrate specificity of a deconjugating enzyme in vitro is vital in understanding the function of this enzyme in vivo, especially when some modifiers or modified proteins exist in trace amounts within the cells.

In human, approximately 100 proteases for deconjugating Ub or Ub chains were found and classified into two main families, cysteine proteases and metalloproteases [7]. The cysteine proteases include six subfamilies, ubiquitin-specific proteases (USPs), ubiquitin C-terminal hydrolases (UCHs), ovarian tumor proteases (OTU), and Machado–Josephin domain proteases (MJDs), while metalloproteases only comprise the Jab1/Mov34/Mpr1 Pad1 N-terminal+ (MPN+) (JAMM) domain proteases, that are zinc-dependent metalloproteinases [8]. Cysteine protease family usually contains a canonical catalytical diad consisting of Cys and His, or triad, including Cys, His, and Asp residues [9]. Despite the fact that varied deubiquitinating enzymes are folded into different catalytic domains, the cysteine residues are almost at the same position to catalyze the deconjugation of the isopeptide bond [9]. These DUBs with distinct spatial structures and catalytic properties accurately regulate diverse cellular processes.

Pathogens have evolved a series of molecular strategies to promote infection, proliferation, and survival during host–pathogen interactions. Many viruses encode viral DUBs to hijack host cell defense mechanisms, such as Herpes simplex virus (HSV), Kaposi’s sarcoma-associated herpesvirus (KSHV), Epstein–Barr virus (EBV), MDV, pseudorabies virus (PRV), Human cytomegalovirus (HCMV), severe acute respiratory syndrome-coronavirus (SARS-CoV), and Middle East respiratory syndrome-related coronavirus (MERS-CoV) which encode USP type DUBs [10,11,12]. Equine arteritis virus (EAV), porcine reproductive and respiratory syndrome virus (PRRSV), Crimean–Congo hemorrhagic fever virus (CCHFV), and Dugbe virus (DUGV) encode OTU type DUBs [13,14]. Regardless of the fact that various viral-derived DUBs regulate the same intracellular target protein or a pathway as their host cell counterpart, they share no structural and sequence homology [15,16]. These differences allow the virus to break through the defense of its host, or avoid the removal of the virus by host cells. Some of these viral enzymes were used as targets of anti-virus drugs. Thus, clarifying the profile of viral DUBs in vitro is very important to the investigation of intracellular functions and regulatory mechanisms of these DUBs.

MDV is an avian alphaherpesvirus that can induce Marek’s disease (MD) in susceptible chickens, which is characterized by multiple organ lymphoma, immunosuppression, and neurological disorders [17]. Although MD could be effectively controlled by vaccines, MD vaccines are incapable of preventing MDV infection, replication, and maturation in chicken cells [17]. With the introduction of MD vaccines in the 1970s, MDV evolved to be even more virulent and remains a threat to the poultry industry [18,19,20]. The mechanism of MDV pathogenesis and virulence enhancement has not been fully elucidated, although specific MDV-encoded genes have been revealed to be important to the MDV pathogenicity [21,22,23,24,25,26,27]. Our previous study found MDV could reverse the ubiquitylome of chicken T-cell lymphoma by comparison with normal CD4^+^ T cells, and the N-terminal 75 kDa fragment of UL36, which contains the viral DUB catalytic domain (UL36-DUB), exists in MDV-induced T lymphoma cells in high level [28]. The amino acid sequence of UL36-DUB is highly consistent throughout all virulent strains of MDV, but not in that of other virus species. It has been reported that mutation of cysteine at the predicted catalytic core of DUB encoded by MDV can significantly reduce the number of chicken T lymphoma [29]; and the region between conserved glutamine (Q85) and the leucine (L106), which includes the active site cysteine (C98), is also required for MDV replication [24]. Therefore, these in vivo investigations have determined that the DUB domain (UL36-DUB) of MDV-encoded large tegument protein plays an important role in MDV replication and pathogenicity, and implied that UL36-DUB could be a potential target for the high-throughput screening of MDV inhibitors in vitro. However, the in vitro preparation method of MDV-encoded UL36-DUB has not been reported, and its deubiquitinating activity and substrate specificity have not been directly confirmed. Although our laboratory has successfully prepared several larger active DUBs previously using various expression strategies, problems on inactivity and insolubility during UL36-DUB preparation were still encountered in the preliminary experiments. In the current study, a soluble and active UL36-DUB was finally prepared using the Bac-to-Bac expression system after multiple optimizations. Herein, the methods for preparing active UL36-DUB, the analysis of its deubiquitinating enzyme kinetics, and substrate specificity are included in this report. Some animal or pathogen DUBs, such as chicken USP1 [30], human USP21 [31], *Legionella pneumophila* SdeA [32], *Plasmodium falciparum* PfUCH54 [33], Epstein–Barr virus BPLF1 [34], reportedly exhibit a de-NEDDylating activity and can de-conjugate NEDD8-modified protein. However, MDV-encoded UL36-DUB shares no amino acid sequence homology with these DUBs. Thus, in the current study, the chicken Ub and NEDD8 inhibitor probes were prepared for the identification of the specificity profile of MDV-encoded UL36-DUB.

## 2. Results

### 2.1. Purification of MDV-Encoded UL36-DUBs

**Figure 1:**
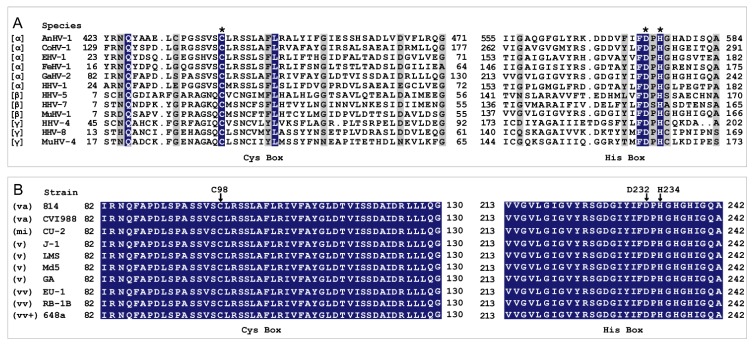
The amino acid sequence alignment between the deubiquitinases (DUBs) encoded by different viruses of *Herpesviridae*. (**A**) Alignment between the DUBs encoded by different species of *Herpesviridae*. (**B**) Alignment between the DUBs encoded by different strains of *Mardivirus*. Asterisks (*) indicate the predicted catalytic triad of viral DUBs. Arrows point to mutation sites. Greek alphabet in square brackets in (**A**) refers to the subfamily of viruses. The abbreviation in parenthesis in (**B**) refers to the virulence of MDV, and va = vaccine; mi = mild; v = virulent; vv =very virulent; vv+ = very virulent plus. (The accession numbers of viral DUBs are given in Tables S2 and S3).

**Figure 2:**
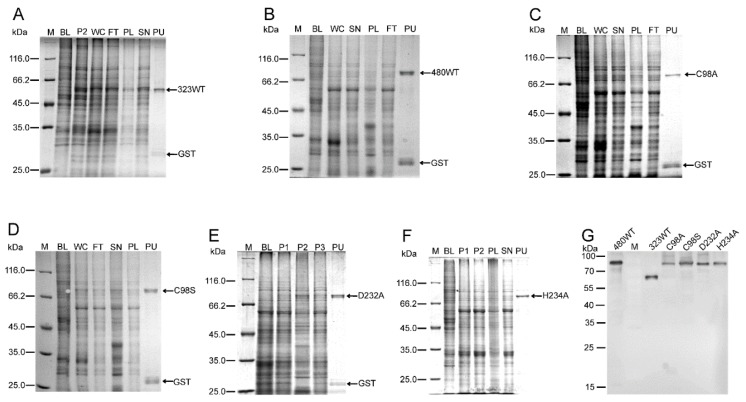
The expression and purification of wild type and mutants of UL36-DUBs. (**A**–**F**), the SDS-PAGE gel images of expression and purification of UL36(323)-GST, UL36(480)-GST, and the mutants. (**G**) A Western blot image of purified UL36-DUBs using the anti-UL36(323) antibody. The lane labels: M refers to protein standard; BL refers to uninfected sf9 cells (blank); WC refers to whole cells expressing target protein; FT refers to the flow-through of the purification column; SN refers to the supernatant of cell lysate; PL refers to the pellet of cell lysate; PU refers to the purified target protein; P1, P2, and P3 refer to sf9 cells used in the preparation of P1, P2, and P3 generation baculovirus, respectively; 480^WT^ refers to wild type UL36(480); 323^WT^ refers to wild type UL36(323); and C98A, C98S, D232A, and H234A refer to UL36(480)^C98A^, UL36(480)^C98S^, UL36(480)^D232A^, and UL36(480)^H234A^ mutant proteins, respectively.

Based on previous reports of MDV- and other virus-encoded DUBs [11,24,29,35,36,37] and analysis of secondary structural integrity, N-terminal 323 and 480 amino acid fragments of MDV-encoded UL36 were investigated in the current study. The amino acid sequence alignment of UL36 catalytic core homologs between 12 representatives of α-, β- and γ- herpesviruses showed an overall low conservation except for the catalytic triad residues, C98, D232, and H234, based on the sequence of UL36 protein of Gallid alphaherpesvirus 2 (GaHV-2) (Figure 1A). However, the amino acid sequence of UL36 remains identical throughout all virulent GaHV-2, MDV, (Figure 1B). In our previous preliminary investigation on the expression of various UL36-DUBs, no soluble or active UL36-DUBs were prepared, even using various vectors or tags to improve folding and solubility in prokaryotic, yeast, insect-baculovirus, or mammalian cell expression systems. Using an optimized strategy (Table S1), both wild type and mutants of UL36-DUB were soluble in the supernatant of cell lysate and were highly pure post purification (Figure 2A–F). The purified UL36-DUBs were detectable by Western blotting using an antibody against UL36(323) (Figure 2G). Although a small amount of GST tag appeared in SDS-PAGE gel (approximately 26 kDa in Figure 2A–F), no truncated UL36-DUBs were detected by Western blotting (Figure 2G), which indicated that the purified UL36-DUBs were intact. As shown in Figure 2, UL36-DUBs were expressed at a very low level. Some types of UL36 proteins were even not visually detectable in the gel image of whole-cell lysate. The expression of UL36-DUBs was at the highest level of sf9 cells infected by P1 generation baculovirus during the preparation of P2 generation baculovirus and then declined sharply at P3 generation such that no UL36-DUBs proteins were visible in the gel image and could be purified from the infected sf9 cells (data not shown). This result suggested that P1 baculovirus is the best generation to infect sf9 for high-level expression, and also implied that UL36-DUBs may be toxic to sf9 cells, and therefore, their expression was inhibited by the sf9 cell protection system. This observation may explain why UL36-DUBs protein could not be found in sf9 cells post recombinant baculovirus infection when routine protocols using other passage viruses other than the P1 virus were employed in the previous efforts.

### 2.2. Wild Type UL36-DUBs Effectively Hydrolyzed Ubiquitin Substrate

To evaluate the deubiquitinating activity of these purified UL36-DUBs, Ubiquitin 7-amido-4-methylcoumarin (Ub-AMC) was used as the substrate for enzymatic kinetic analysis (Table 1). The results showed that wild type UL36(323) and UL36(480) exhibited deubiquitinating activity. The catalytic ability of UL36(323) was slightly lower than that of UL36 (480) but not significantly different. This result indicated that the flanking sequence of the catalytic core may be conducive to the deubiquitinating activity of UL36. According to the sequence alignment of various viral DUBs (Figure 1) and literature [24], the predicted catalytic triad was mutated. The enzyme kinetics results suggested that the deubiquitinating activity was derived from UL36-DUB itself rather than the protease, which might be introduced during the purification process. The mutants, UL36(480)^C98A^, UL36(480)^C98S^, and UL36(480)^H234A^, were not able to hydrolyze Ub-AMC substrate, while the mutant UL36(480)^D232A^ exhibited a weaker deubiquitinating activity than wild type UL36-DUBs (Table 1). This result suggested that the two residues, C98 and H234, are essential for UL36-DUB activity, and D232 contributes to UL36 deubiquitinating activity but is not indispensable. This kinetics result further confirmed that the strategy described in this report was effective for obtaining soluble and active UL36-DUBs.

### 2.3. UL36 Hydrolyzed Ubiquitin Chains in Linkage Preference

**Figure 3:**
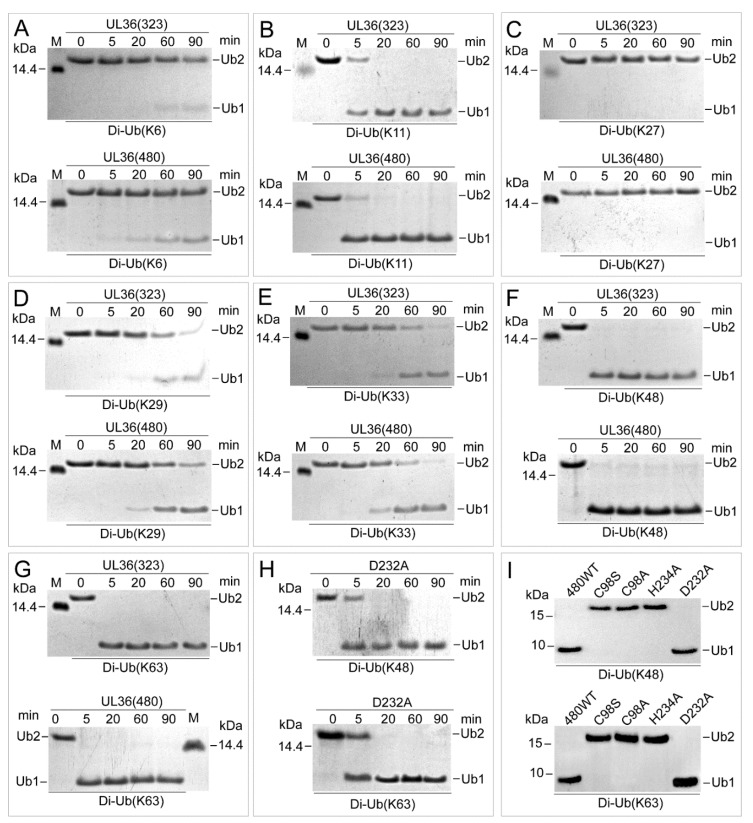
Characterization of ubiquitin (Ub) linkage preference of UL36(323) and UL36(480). (**A**–**G**) SDS-PAGE images of UL36-DUBs digestion products on various types of dimeric Ub chains. (**H**) SDS-PAGE images of the products of UL36(480)^D232A^ hydrolyzing K48 and K63 linkage Ub chains. (**I**) Western blotting of digestion products of wild type 36 on K48 and K63 linkage Ub chains after a 90-min incubation. The labels under each image indicate different types of Ub linkage dimer. Labels on the sides of each picture represent different lengths of Ub; Ub2, Ub dimer; Ub1, Ub monomer. The numbers (0, 5, 20, 60, and 90) on the top of the images (**A**–**H**) indicate the incubation time in minutes. In (**H**,**I**), 480^WT^, wild type UL36(480); C98S, C98A, H234A, and D232A indicate UL36(480)^C98S^, UL36(480)^C98A^, UL36(480)^H234A^, and UL36(480)^D232A^ mutants, respectively. M in images (**A**–**G**) labels the protein standard.

**Figure 4:**
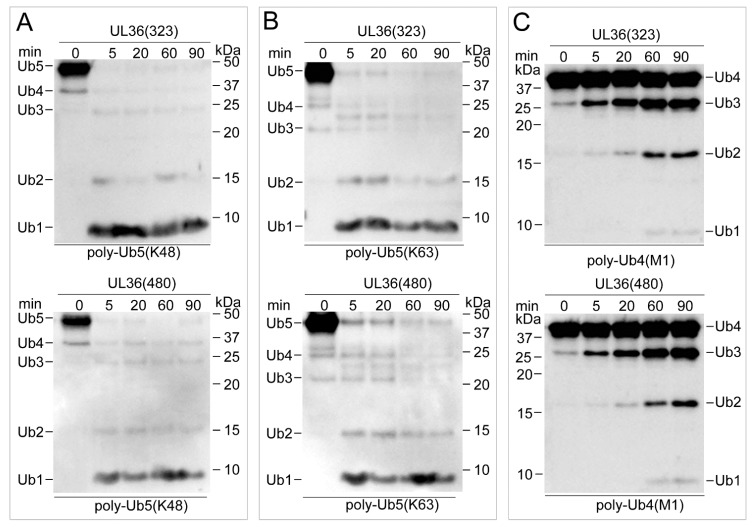
Characterization of substrate preference of UL36(323) and UL36(480) using poly-Ub chains. (**A**–**C**) The identification of UL36-DUBs digestion products on various types of poly-Ub chains by Western blotting. The labels under each image indicate different linkage types of Ub polymer. Labels on the sides of each image represent different lengths of Ub; Ub1, Ub monomer; Ub2, Ub dimer; Ub3, Ub trimer; Ub4, Ub tetramer; Ub5, Ub pentamer; M1, linear type of polyubiquitin chain. The numbers on the top of images indicate the incubation time, 0, 5, 20, 60, and 90 min.

To characterize the hydrolysis efficiency of recombinant UL36-DUBs on Ub substrates, various linkage types of Ub dimers and polymers were used as substrates. Hydrolysis results showed that both wild types of UL36-DUBs hydrolyzed various types of Ub chains at different efficiency, but no significant differences were found between the wild type of UL36(480) and UL36(323) on the same substrate (Figure 3 and Figure 4). They preferred to hydrolyze K11, K48, and K63 linkage Ub chains. They were able to completely cleave the K11 types Ub dimers after 20 min, K48 and K63 dimers after 5 min of incubation (Figure 3B,F,G). When comparing the hydrolysis activity of wild type UL36(480) to that of mutants on K48 and K63 dimer (Figure 3H,I), the mutant, UL36(480)^D232A^, was able to turn over the K48 and K63 types of Ub dimers to monomer completely after 20 min of incubation, while UL36(480)^C98A^, UL36(480)^C98S^, and UL36(480)^H234A^ were not able to hydrolyze K48 and K63 type Ub dimers after 60 min of incubation. Combined with the kinetics result, it confirmed that C98 and H234 were the key residues for UL36-DUB activity and suggested that the mutation on D232 residue did slightly impact UL36 catalytic efficiency. The wild type UL36-DUBs could partially hydrolyze K6, K29, and K33 Ub chains, but there was no activity on K27 Ub chains (Figure 3A,C–E). Compared to Ub dimer hydrolysis results, wild type UL36-DUBs were not able to hydrolyze Ub polymer substrates completely, even after 90 min of incubation (Figure 4). The efficiency of UL36-DUBs in hydrolyzing K48 and K63 types of Ub chains was higher than that on the linear type of Ub chains (M1) (Figure 4). According to these results, the proteins modified by K11, K48, and K63 Ub chains might be the preferred targets of MDV-encoded UL36 in vivo.

### 2.4. UL36-DUB Failed to Deconjugate UbL Substrates

**Figure 5:**
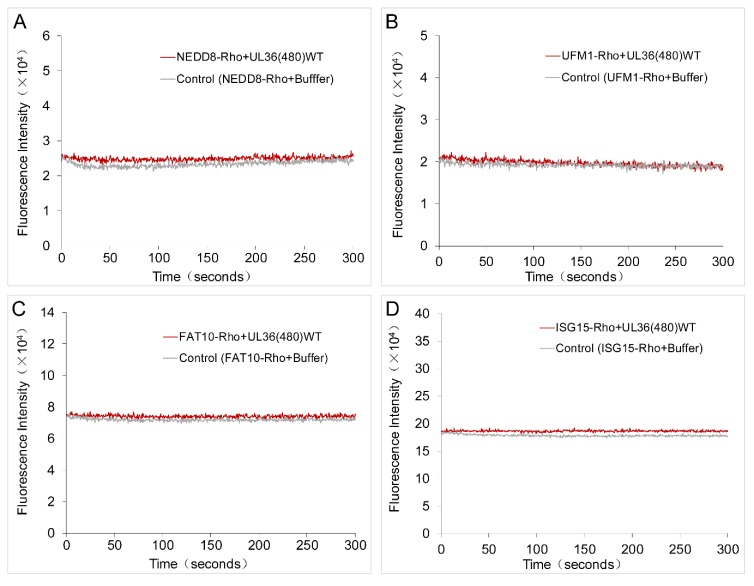
Characterization of hydrolysis activity of UL36(480)^WT^ on rhodamine-conjugated Ub-like substrates. (**A**–**D**), fluorescence traces of rhodamine released on the hydrolysis of rhodamine-conjugated NEDD8 (**A**), UFM1 (**B**), FAT10 (**C**), and ISG15 (**D**) substrates by wild type UL36(480), UL36(480)^WT^. Control, the group contained an equal volume of buffer but no UL36(480)^WT^.

**Figure 6:**
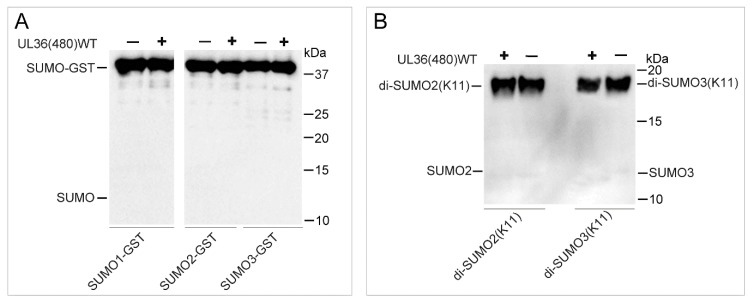
Identification of UL36(480)^WT^ specificity using SUMO substrates. (**A**) Western blot identification of UL36(480)^WT^ digestion products using substrates SUMO-GST after a 90-min incubation; (**B**) Western blot identification of UL36(480)^WT^ digestion products using substrates K11 linkage type of di-SUMO2/3 after a 90-min incubation. -, absence of UL36(480)^WT^ in reaction system; +, addition of UL36(480)^WT^ in the reaction system.

Although various UbL proteins share a higher structure similarity to Ub, and some enzymes show cross-reactivity on Ub and NEDD8 substrates, most of the deconjugating enzymes for these modifiers exhibit strict hydrolysis selectivity on Ub and UbL substrates. To determine whether UL36-DUBs have cross-reactivity on these UbL modifiers, SUMO1, SUMO2, SUMO3, NEDD8, FAT10, ISG15, and UFM1 dimer or rhodamine-conjugated substrates, and the inhibitor probes, Ub-Br and NEDD8-Br, were applied in an analysis of substrate preference. The fluorescence results showed that the Rho fluorophore could be released from Ub-Rho substrates (Figure S1), but not from UbL-Rho substrates (Figure 5), compared to the respective control group, which indicated that UL36(480)^WT^ was not able to hydrolyze NEDD8, UFM1, FAT10, and ISG15 substrates (Figure 5). In addition, the fluorescence results also showed that there were large differences in the fluorescence signal background of different substrates in negative control groups, and this was most likely due to the different Rho remaining in the production process. However, this phenomenon did not affect comparability between the reactions using the same substrate. The results from binding assays showed that UL36(480)^WT^ was able to bind to the Ub-Br probe but not the NEDD8-Br probe. As for SUMO substrates, UL36(480)^WT^ enzyme was not able to hydrolyze GST tagged SUMO1, SUMO2, and SUMO3 substrates and K11 linkage type of SUMO2 and SUMO3 substrates (Figure 6). These results suggested that UL36(480)^WT^ enzyme may possess a strict de-conjugating activity on Ub substrates but not cross-reactivity on UbL modifiers. These results strongly indicated that ubiquitinated proteins were the specific targets of UL36 encoded by MDV.

### 2.5. UL36 Specifically Targets Ub but Not NEDD8

**Figure 7:**
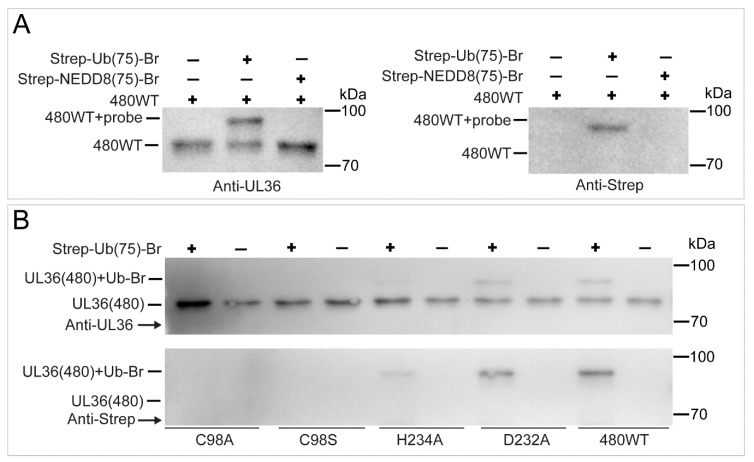
Identification of UL36-DUBs binding to the Strep-Ub(75)-Br or Strep-NEDD8(75)-Br probe. (**A**) Identification of the products of UL36(480)^WT^ binding to the Strep-Ub(75)-Br and Strep-NEDD8(75)-Br probes by Western blot using anti-UL36 (left panel) or anti-strep (right panel) antibodies; (**B**) Identification of the products of UL36(480)^WT^ and mutants binding to the Strep-Ub(75)-Br probe by Western blot using anti-UL36 (upper panel) or anti-strep (lower panel) antibodies. 480^WT^, wild type UL36(480); C98S, C98A, D232A, and H234A refer to UL36(480)^C98S^, UL36(480)^C98A^, UL36(480)^D232A^, and UL36(480)^H234A^ mutants, respectively. Probe, Strep-Ub(75)-Br or Strep-NEDD8(75)-Br probe.

**Figure 8:**
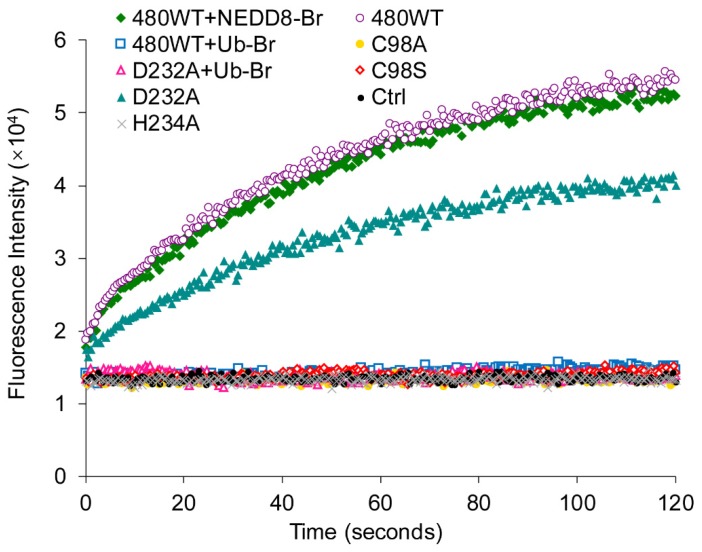
Identification of the catalytic activity of UL36 after incubation with probes. The markers in the scatter chart indicate fluorescence intensity of 7-amido-4-methylcoumarin (AMC) fluorophore released from the reaction catalyzed by various UL36-DUBs proteins using Ub-AMC substrate. 480^WT^, wild type UL36(480); C98S, C98A, D232A, and H234A refer to UL36(480)^C98S^, UL36(480)^C98A^, UL36(480)^D232A^, and UL36(480)^H234A^ mutants, respectively; Ctrl (black dot), the reaction without addition of any UL36-DUB; Ub-Br, Strep-Ub(75)-Br probe; NEDD8-Br, Strep-NEDD8(75)-Br probe.

To further characterize the substrate specificity of UL36-DUBs, a Strep-tagged Ub-Br probe was used to bind to UL36-DUBs, and a Strep-tagged NEDD8-Br probe was used to identify the cross-reactivity of UL36-DUBs. As shown in Figure 7, wild type UL36(480) bound to the Ub-Br probe but not the NEDD8-Br probe. The UL36(480) mutants, UL36(480)^D232A^ and UL36(480)^H234A^, bound to the Ub-Br probe at a lower level, but not the Cys98 mutants, UL36(480)^C98S^, and UL36(480)^C98A^ (Figure 7A,B). The mutants, UL36(480)^C98A^ (gold dot), UL36(480)^C98S^ (hollow red diamond). and UL36(480)^H234A^ (gray cross), were not able to hydrolyze the Ub-AMC substrate (Figure 8). The ability of UL36(480)^D232A^ mutant proteins (cyan triangle) to hydrolyze Ub-AMC was lower than that of UL36(480) wild type (hollow purple circle). UL36(480) wild type (hollow blue square) and UL36(480)^D232A^ mutant proteins (hollow pink triangle) that bound to the Ub-Br probe failed to hydrolyze the Ub-AMC substrate (Figure 8). These results indicated that C98 may be the catalytic residue of UL36-DUB deubiquitinating activity. In addition, UL36(480) wild type did not bind to the NEDD8-Br probe (Figure 7A). The NEDD8-Br probe did not disrupt UL36 deubiquitinating activity (green diamond in Figure 8). This finding suggested that UL36-DUB has no cross-reactivity on the NEDD8 substrate. This result was consistent with the hydrolysis result (Figure 5).

### 2.6. UL36-DUBs Was Resistant to Some Protease Inhibitors

**Figure 9:**
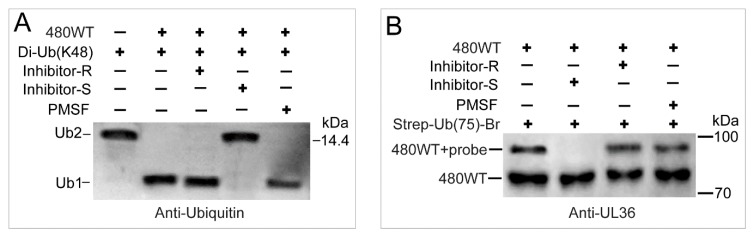
The interference of inhibitor on the activity and binding of UL36(480) wild type protein. (**A**) The inhibition of inhibitors on the deubiquitinating activity of UL36(480) wild type on di-Ub(K48) substrate. (**B**) The inhibition of inhibitors on the binding of probes to UL36(480) wild type protein. 480^WT^, wild type UL36(480); Inhibitor-R, Roche inhibitor cocktail; Inhibitor-S, Solarbio inhibitor cocktail; PMSF, phenylmethanesulfonyl fluoride, a serine protease inhibitor; Ub2, Ub dimer; Ub1, Ub monomer.

Protease inhibitors were tested for the inhibition of the deubiquitinating activity of UL36-DUB. As shown in Figure 9A, phenylmethanesulfonyl fluoride (PMSF), a serine protease inhibitor, at 1 ×, even up to 5 ×, working concentration was tested but failed to inhibit UL36-DUB activity. Roche inhibitor cocktail (Complete protease inhibitor cocktail, CPIC), a protease inhibitors mixture including cysteine protease inhibitors, did not inhibit the DUB activity of UL36 at 1 × working concentration (Figure 9A) and failed to completely inhibit even when the concentrations were up to 5 times (data not shown). One times working concentration of the Solarbio inhibitor cocktail did inhibit the DUB activity of UL36 and its binding to the Ub-Br probe (Figure 9B). This result confirmed that UL36 is of Cys protease activity and suggested that PMSF and Roche CPIC could be used in UL36-DUB purification or pull-down assay.

## 3. Discussion

The ubiquitin regulation system is one of the most important regulating systems and the target of many pathogens in cells. The cellular reversible ubiquitination regulatory machinery consisting of ubiquitinating cassette and deubiquitinating enzymes can change intracellular homeostasis to modulate cell fate [1]. Modifiers involved in these regulations include monomers of ubiquitin and UbL, homopolymeric and heteropolymeric chains, in each of which the modifier complexly and specifically regulates different cell activities and leads to distinct cell fates [15]. Viruses also encode some viral types of modifiers, relevant catalytic enzymes, or regulatory proteins to mimic the counterparts of the host cell but sharing no homology in amino acid sequence [15]. This feature allows viruses and their encoded products to avoid recognition and elimination by host cells, thereby promoting the pathogenicity and survival of viruses [16]. Thus, revealing the characteristics and functions of virus-encoded products should help to advance the understanding of pathogenic mechanisms, and these viral products, in turn, could potentially be used as targets for the development of new antiviral drugs. However, it is difficult to directly analyze the properties of these modifiers or enzymes in situ and in real-time if the existence of those modifiers and/or enzymes were in a small amount or had a short half-life during cellular processes. Isolation of active and soluble DUBs in vitro is the key step for such a study. MDV-encoded DUB, UL36, shares no homology with any other types of DUBs. It has been reported that the mutation of the key residues in the predicted catalytic core impacts MDV replication, tumorigenicity, and breeding, which indicates that UL36-DUB is important to the pathogenicity of MDV [24,29]. To prepare the UL36-DUB for the investigation of the substrate specificity of UL36 (a giant protein of ~ 367 kDa), several gene fragments encoding different lengths of the UL36 fragments (36~113 kDa) were subcloned into eukaryotic and prokaryotic vectors for expression and purification in our preliminary work. However, the soluble and active UL36-DUB proteins were not able to be prepared using varied vectors, such as the prokaryotic vectors directed by T7 or Arab promoters, the baculoviral expression vector directed by pH or p10 promoter in varied insect cells, and yeast expression vectors directed by AOX and other promoters, even though different tags and expression cells were used. Summarizing the previous experience, the UL36-DUBs genes were optimized, and GST was used as a solubility-promoting tag to be fused on C-terminus of UL36-DUBs to mimic C-terminal fragment of UL36. The generation of recombinant baculovirus was also optimized for increasing the expression level of UL36-DUBs’ proteins. Eventually, the soluble and active UL36-DUBs were prepared, although the expression rate in whole cells was very low, and even the expression band was absent in P3 generations of infected sf9 cells. This observation implied that UL36-DUBs may be toxic to insect cells, and may trigger the self-protection mechanism of sf9 cells to cause the expression decrease or degradation of UL36.

UL36-DUBs prepared in the current study showed linkage-dependent activity, preferably hydrolyzing K11, K48, and K63 ubiquitin chains. The K11 or K48 type Ub chain modification is mainly involved in the proteasome degradation of the modified protein, while the K63 type Ub chain modification regulates the function of the modified protein [38]. In addition, MDV can reverse the ubiquitination that relates to the infection, inflammation, tumor, and immune-related proteins in T lymphocyte tumors [28]. These observations indicated that the cellular proteins modified by these three types of Ub chains should be the targets of MDV-encoded UL36. UL36 may deubiquitinate these target proteins to regulate the apoptosis of B cells and the transformation of T cells induced by MDV. A substrate preference assay revealed that UL36 strictly targeted the modification by ubiquitin but not by UbL. These results suggested that UL36 may not regulate the pathways participated by UbL modification in MDV-infected cells, implying that the ubiquitin regulation plays an important role in MDV pathogenicity.

MD in chickens is a serious threat to the poultry industry characterized by immunosuppression, multiple organ lymphoma, and mortality. The MD vaccine can control the occurrence of MD, but cannot prevent the infection and replication of MDV, or solve the problems of MDV virulence increasing year by year [19,20,26,39,40]. Virulent MDV can cause acute death in unimmunized susceptible chickens within a few days after infection. The vaccine immunization extends the life of the MDV-infected chickens and, on a negative effect side, provides sufficient time for MDV proliferation, maturation, and spreading [17,19,20,26,39,40]. This process was thought to be one of the main factors, which facilitates the enhancement of MDV virulence [19,20,26,39,40]. Therefore, choking the pathway of MDV intracellular activity may be an effective strategy for stifling virus virulence enhancement and for improving vaccine immune efficacy. There are virus-encoded DUBs that have been used to develop antiviral drugs, such as the two DUBs encoded by SARS-CoV and MERS-CoV. The inhibitors of the two enzymes can specifically resist the two viruses, respectively, and there is no cross-reactivity [41]. The activity of HIV-encoded protease, HIV-1, is critical for the packaging and infectivity of HIV [42]. HIV-1 enzymes have been targeted for the screening of anti-HIV drugs, among which specific inhibitors, saquinavir and ritonavir, have been approved to be used in clinical trials [43,44]. UL36 is highly conserved in different virulent MDVs, indicating that the activity of UL36 is evolutionarily important to the pathogenicity and replication of MDV, and the UL36 catalytic domain could be used as a target to develop the drug for curbing the increasing virulence and pathogenicity of MDV. This study provided a tested protocol for the preparation of an active UL36-DUB and characterized the properties of UL36-DUB, which should be highly advantageous to future investigations on high-throughput screening of UL36-DUB inhibitors in vitro for development of anti-MDV reagents.

In the process of protein purification, the mixture of various protein inhibitors, such as PMSF, Roche CPIC, and Solarbio inhibitor cocktail, are often used to inhibit various proteases in the cell lysate. Some DUBs prepared in our previous study were sensitive to protease inhibitors, while MDV-encoded UL36 is resistant to some inhibitors. For example, PMSF and Roche CPIC did not inhibit the activity of UL36 from a working concentration up to 5 × concentration, but the Solarbio inhibitor cocktail did. In addition, the UL36-DUB exists in MDV-induced T lymphoma cells for a long term [28], and chicken cells did not wipe off this foreign enzyme that seriously affects the intracellular environment promoting MDV activity. These observations indicated that UL36-DUB may have strong anti-antagonism and anti-proteolysis characteristics, and could be kept intact in host cells. It has been observed that UL36 was susceptible to proteolytic degradation during the lysis of MDV-induced T lymphoma cells in our previous study [28]. Thus, PMSF and Roche CPIC could be used in cell lysis during the survey of the function of UL36 to protect UL36 from degradation, as well as to avoid interference with UL36 activity.

Cys residue in the catalytic core of the cysteine protease DUB family is the most important site for deubiquitinating activity. His residue in the catalytic core provides a nucleophilic attack circumstance to Cys and then lowers the pKa of the Cys, promoting the occurrence of dissociation on the isopeptide bond between the ubiquitin C-terminus and the substrate [9]. Thus, determining these key residues in DUB is very important to the investigation of the function and property of DUB. In vivo study has elucidated that the mutation on C98 residue in the catalytic core of UL36 inhibits the replication of the MDV genome, decreases the incidence of MD tumors, and reduces the pathogenicity and the horizontal transmission of MDV [24,29]. The current in vitro study found that the mutations on C98 and H234 could eliminate the deubiquitinating activity of UL36-DUB, but not on D232 residue. These observations from the in vivo and in vitro tests determined that C98 and H234 residues are indispensable to the deubiquitinating activity of UL36. In addition, although the mutation of D232 was observed with a slight impact on UL36 catalytic efficiency, it might be potentially attributable to an alteration of the charge environment of the catalytic core or the spatial structure of UL36-DUB due to the replacement of charged amino acids by non-polar amino acids. This mutation result in the current study implied that the catalytic core of UL36-DUB may consist of a diad but not triad because the D232 residue was not indispensable to UL36-DUB activity.

In summary, this study revealed the substrate preference of MDV-encoded UL36-DUB and provided a feasible protocol for obtaining soluble and active UL36-DUB. The properties elucidated in this study should promote further investigations for the insights in advancing the basic understanding of UL36 functions in genomic integration, tumorigenesis, and immunosuppression. These findings have also laid the foundation for future screening of anti-MDV drugs with UL36-DUB as a target, as well as for research to investigate even longer UL36 fragments in vitro.

## 4. Materials and Methods

### 4.1. Construction of Plasmids

**Figure 10:**
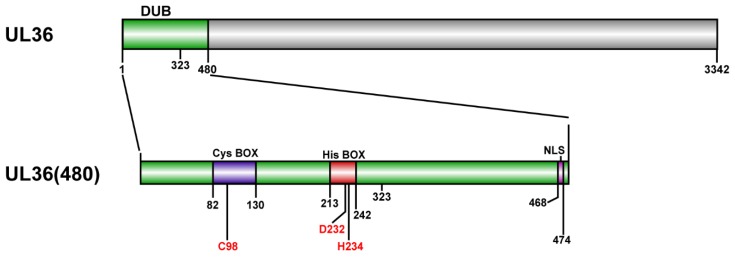
Diagram of UL36 encoded by Marek’s disease virus (MDV). Top chart indicates the full length of UL36; lower chart indicates UL36(480) fragment. The numbers are sites of the residues on UL36, where numbers in red are the predicted catalytic triad. DUB, the predicted DUB domain of UL36; NLS, nuclear localization sequence.

According to UL36 gene encoded by virulent MDV strain J-1 genome (GeneBank ID KU744555) [45] and insect genetic code (*Spodoptera frugiperda*), the gene of *UL36(480)* (Diagram in Figure 10 that was generated in software DOG 2.0 [46]), which contains a 480 amino acid N-terminal sequence of UL36, was optimized on codon usage bias, GC-content, mRNA secondary structure, repeat sequences, restriction enzyme recognition sites, using software Codon OptimWiz (Genewiz Inc. South Plainfield, NJ, USA) (https://www.genewiz.com/en/Public/Services/Gene-Synthesis/codon-optimization) (Table S1). For expression with the Bac-to-Bac system, Sal I and Hind III were fused on the up- and down-stream of the *UL36(480)* gene, respectively. The gene of *GST tag* was amplified using primers GST-F-Hind III and GST-R-Pst I from pGEX-4t-3 plasmid (GE Healthcare Lifesciences, Pittsburgh, PA, USA) by PCR. *UL36(480)* and *GST tag* genes were subcloned into pFast-Bac-Dual with Sal I, Hind III, and Pst I to construct pFast-Bac-Dual-UL36(480)-GST donor plasmid. The *UL36(323)* gene was amplified using an optimized *UL36(480)* gene as a template and U-F-Sal I and U323-R-Hind III as primers, then subcloned into pFast-Bac-Dual with Sal I, Hind III, and Pst I to construct pFast-Bac-Dual-UL36(323)-GST donor plasmid. The recombinant plasmid pFast-Bac-Dual-UL36(480)-GST was used as a template to generate C98A, C98S, D232A, and H234A mutants of UL36(480), respectively, in the putative catalytic core mutants using a QuikChange Site-Directed Mutagenesis kit (Agilent Tech. Inc., Santa Clara, CA, USA) according to the manufacturer’s instructions, with respective primers, C98A-F and C98A-R, C98S-F and C98S-R, D232A-F and D232A-R, H234A-F and H234A-R, listed in Table 2.

### 4.2. Expression and Purification of UL36-DUBs Proteins

DH10Bac competent cells were transformed, respectively, with recombinant donor plasmids, pFast-Bac-Dual-UL36(323), pFast-Bac-Dual-UL36(480), and pFast-Bac-Dual-UL36(480) mutants, UL36(480)^C98A^, UL36(480)^C98S^, UL36(480)^D232A^, and UL36(480)^H234A^, to generate the recombinant bacmids. According to the manual of the Bac-to-Bac expression system (Invitrogen Corporation, Carlsbad, CA, USA), sf9 cells were transfected by the recombinant bacmids to generate recombinant baculovirus, followed by the expression of UL36-DUBs proteins through infecting sf9 cells with recombinant baculoviruses. Sf9 cells expressing the proteins of interest were ultrasonicated in lysis buffer (50 mM Tris HCl pH 8.0, 5% glycerol). Insoluble material was removed by centrifugation at 15,000× *g* for 1 h at 4 °C. The harvested supernatant was filtrated through a 0.22-μm filter, followed by loading onto a Glutathione agarose column (Thermo Fisher Scientific, Waltham, MA, USA). Post extensive wash with lysis buffer, the proteins bound on the column were eluted using an elution buffer (50 mM Tris HCl pH 8.0, 5% glycerol, and 25 mM reduced glutathione). The eluted proteins were dialyzed against a reaction buffer (50 mM HEPES-KOH pH 7.8, 150 mM NaCl, 0.1 mg/mL BSA, 0.5 mM EDTA, and 1 mM DTT). Purified protein aliquots were either used immediately or flash-frozen in liquid nitrogen and stored at −80 °C.

### 4.3. Investigation of the Kinetics of Deubiquitinating Activity

The kinetics of the deubiquitinating activity of wild type UL36(323), UL36(480), and the mutants, UL36(480)^C98A^, UL36(480)^C98S^, UL36(480)^D232A^, and UL36(480)^H234A^, were tested using Ub-AMC substrate. The reactions containing 2 nM UL36(323), UL36(480) or UL36(480)^D232A^, or 200 nM other UL36(480) mutants, 0.1 to 1 μM Ub-AMC were performed in 1× reaction buffer. The deubiquitinating activity of each UL36-DUB type was assessed by measuring the fluorescence intensity of the released AMC on a FluoroMax 4 fluorescence spectrophotometer (HORIBA Scientific) with excitation and emission wavelengths of 380 nm and 460 nm, respectively. The enzyme kinetics were calculated by plotting the reaction velocity versus the substrate concentration in GraphPad Prism 8 software (GraphPad Software Inc., La Jolla, CA, USA). A Michaelis–Menten plot in GraphPad Prism 8 was used to fit the curves using the equation V = (V_max_• [S])/([S] + *K*_M_). A Lineweaver–Burk plot was used to determine the *K*_M_ and V_max_, and then the V_max_/enzyme concentration to calculate the catalytic constant *k*_cat_. The catalytic efficiency of each type of UL36 was compared using the ratio *k*_cat_/*K*_M_.

### 4.4. Characterization of Ub Substrate Preference of UL36-DUBs

The substrate preference of wild type UL36(323), UL36(480), and the mutants, UL36(480)^C98A^, UL36(480)^C98S^, UL36(480)^D232A^, and UL36(480)^H234A^, was characterized by testing the hydrolysis efficiency on a variety of Ub substrates. A reaction system containing 98 μM of substrate (K6-, K11-, K27-, K29-, K33-, K48-, K63-, or M1- linked ubiquitin chain (Boston Biochem, Cambridge, MA, USA), 1× reaction buffer in a final volume of 100 μL was initiated by adding 200 nM UL36-DUBs at 37 °C for different incubation times. The hydrolyzed products were separated by SDS-PAGE and stained with Coomassie brilliant blue R-250. Standard Western blotting was performed using primary antibody against ubiquitin (Cat#: ab7780, dilution 1:2000, Abcam, Cambridge, MA, USA), and horseradish peroxidase (HRP)-conjugated goat anti-rabbit IgG secondary antibody (Cat#: TA130023, dilution 1:5000, OriGene, Rockville, MD, USA) was used for ECL imaging.

### 4.5. Characterization of the UbL Substrate Specificity of UL36

SUMO1-GST, SUMO2-GST, SUMO3-GST [47,48], di-SUMO2(K11), or di-SUMO3(K11) and various rhodamine-conjugated ubiquitin-like proteins (Boston Biochem) were used as substrates for the characterization of hydrolysis specificity of 1 μM UL36(480). The reactions were performed as described above using SUMO substrate, and hydrolysis products were identified by Western blotting using primary antibody against SUMO1 (Cat#: ab5316) or SUMO2/3 (Cat#: ab3742) (dilution 1:2000, Abcam), and HRP-conjugated goat anti-rabbit IgG secondary antibody (1:5000, OriGene) was used for ECL imaging. Hydrolysis specificity of UL36(480) on rhodamine-conjugated ubiquitin-like proteins was assessed in reactions containing 0.1μM of FAT10-Rhodamine, NEDD8-Rhodamine, UFM1-Rhodamine, or ISG15-Rhodamine substrates (Boston Biochem), respectively, and 1 μM UL36(480) in a final volume of 100 μL 1× reaction buffer at 37 °C. The fluorescence intensity of rhodamine group hydrolyzed from the reactions was monitored on a FluoroMax 4 fluorescence spectrophotometer (HORIBA Scientific, Edison, NJ, USA) with excitation and emission wavelengths of 570 and 590 nm, respectively.

### 4.6. Preparation of Inhibitor Probe

Some animal or pathogen DUBs have dual deubiquitinating/de-NEDDylating activity [30,31,32,33]. Although the key residues of UL36-DUB are highly conserved compared with other USP type DUBs, UL36-DUB shares no homology on amino acid sequence with these DUBs. To reveal whether MDV-encoded UL36-DUB hydrolyze chicken NEDD8, the irreversible inhibitor probes, Strep TagII-tagged chicken Ub-Br and NEDD8-Br, as active site-specific probes were prepared according to the protocol [49,50]. In brief, referring to the gallus *ubiquitin* gene (GenBank ID M14693.1) and the *NEDD8* gene (GenBank ID XP_015130268.1), the sequence of two genes were optimized using software Codon OptimWiz (Genewiz Inc.). The genes of chicken Ub(75) and NEDD8(75) proteins from which the Gly76 of chicken Ub(76) and NEDD8(76) were absent were amplified by PCR with primers, Ub-probe-F and Ub-probe-R, and NEDD8-probe-F and NEDD8-probe-R (Table 2), and then subcloned into a pTYB1 vector with restriction enzymes (Nde I and Sap I). Ub(75) or NEDD8(75) was fused to a N-terminal Strep-Tag-II tag and a C-terminal intein-CBD tag (intein fused Chitin binding domain on the vector). The fusion protein Strep-Ub(75)-intein-CBD or Strep-Nedd8(75)-intein-CBD was expressed in *E. coli* BL21 (DE3) with the induction by 0.1 mM isopropyl-β-d-thiogalactoside (IPTG). The whole purification process was performed according to the manual of IMPACT™-CN System (New England Biolabs, Ipswich, MA, USA), except that the elution was carried out post the incubating column with cleavage buffer (20 mM Tris-HCl pH 8.5, 1 M NaCl, 1 mM EDTA, 50 mM mercaptosulfonate sodium salt (MESNa)) for 12 h at 25 °C to generate Strep-Ub(75)-MESNa or Strep-Nedd8(75)-MESNa. Strep-Ub(75)-MESNa or Strep-Nedd8(75)-MESNa was incubated with 400 mM of bromoethylamine at pH 8.0 for 20 min at 25 °C to generate inhibitor probe, Strep-Ub(75)-Br or Strep-Nedd8(75)-Br. Finally, the prepared probe was dialyzed in a storage buffer (137 mM NaCl, 2.7 mM KCl, 10 mM Na_2_HPO_4_, 2 mM KH_2_PO_4_, 10% (*v*/*v*) glycerol, pH 7.5), aliquoted for use or stored at −80 °C.

### 4.7. Determination of the Cross-Reactivity of UL36-DUB via Site-Specific Incorporation

For the direct binding test, 5 pmol of the Strep-Ub(75)-Br or Strep-Nedd8(75)-Br probe was incubated with 4 pmol of UL36(480) for 1 h at 37 °C. The reaction products were separated with SDS-PAGE and identified by Western blotting using standard protocols [30] with primary antibody rabbit anti-Strep (Cat#: ab76949, Abcam, Cambridge, MA, USA) or anti-UL36(323) [51] antibody at a dilution of 1:4000 and HRP-conjugated goat anti-rabbit IgG secondary antibody at a dilution of 1:5000 (OriGene Technologies).

For the catalytic activity assay of the probe treated UL36(480), 125 nM UL36(480) or mutants, and 4 μM Strep-Ub(75)-Br or Strep-Nedd8(75)-Br were preincubated in 1× reaction buffer for 1 h at 37 °C, in this case, a final concentration of 100 nM Ub-AMC was added to initiate the deubiquitinating reaction. The deubiquitinating activity was evaluated by measuring the fluorescence intensity of the AMC released from the reaction on a FluoroMax 4 fluorescence spectrophotometer (HORIBA Scientific) with excitation and emission wavelengths of 380 nm and 460 nm, respectively.

For the assay of inhibitor interference to the binding between probes and UL36(480), UL36(480) protein was pre-incubated with 1× working concentration (1 mM) of phenylmethanesulfonyl fluoride (PMSF) (Cat#: 78830, Millipore Sigma, St. Louis, MO, USA) and two types of protease inhibitor cocktails, respectively, manufactured by Solarbio Lifesciences (Cat#: P6730, Tongzhou District, Beijing, China) or Roche of Millipore Sigma (Cat#: 04693116001, St. Louis, MO, USA) for 1 h at 4 °C, and then followed by the catalytic activity assay as described above. Western blotting was carried out to assess the inhibition effect using primary antibodies against ubiquitin, as described above. Two hundred millimolar stock solution of PMSF was freshly dissolved in DMSO, and the stock solutions of both inhibitor cocktails were prepared fresh before use according to the instructions.

## Figures and Tables

**Table 1:** A summary of results in kinetic analysis of UL36-DUBs.

UL36-DUB Proteins	*K*_M_ (μM)	V_max_ (nM/s)	*k*_cat_ (s^−1^)	*k*_cat_/*K*_M_ (mM^−1^s^−1^)	R^2^
UL36(323)^WT^	1.180 ± 0.1490	1.693 ± 0.1323	0.847 ± 0.1358	717.797 ± 60.859	0.99
UL36(480)^WT^	1.0554 ± 0.1262	1.724 ± 0.1233	0.862 ± 0.0682	816.752 ± 52.949	0.99
UL36(480)^D232A^	2.444 ± 0.611	2.747 ± 0.5190	1.374 ± 0.263	561.99 ± 28.448	0.99

**Table 2:** Primers for construction and mutation.

Primer Name	Primer Sequence
U-F-Sal I	5’-ATACGCGTCGACATGACTGACAGCACTGAC-3’
U480-R-Hind III	5’-ATTCCCAAGCTTGCTCTGGGGGGTCCAGAG-3’
U323-R-Hind III	5’- ATTCCCAAGCTTGGGGTCGAAGTCAGCGGACA -3’
GST-F-Hind III	5’-ATTCCCAAGCTTCTGGTTCCGCGTGGATCCATG-3’
GST-R-Pst I	5’-AGTGCACTGCAGCTGGTTCCGCGTGGATCCATG-3’
C98A-F	5’-TGCTTCCTCCGTGTCC**gc**CCTGCGTTCCTCCCTCGCTTTC-3’
C98A-R	5’- GAAAGCGAGGGAGGAACGCAGG**gc**GGACACGGAGGAAGCA-3’
C98S-F	5’- TGCTTCCTCCGTGTCCTcCCTGCGTTCCTCCCTCGCTTTC -3’
C98S-R	5’- GAAAGCGAGGGAGGAACGCAGG**g**AGGACACGGAGGAAGCA -3’
D232A-F	5’-CGACGGTATCTACATCTTCG**c**CCCTCACGGCCACGGCCAC-3’
D232A-R	5’-GTGGCCGTGGCCGTGAGGG**g**CGAAGATGTAGATACCGTCG-3’
H234A-F	5’- GTATCTACATCTTCGACCCT**gc**CGGCCACGGCCACATCGGC -3’
H234A-R	5’- GCCGATGTGGCCGTGGCCG**gc**AGGGTCGAAGATGTAGATAC -3’
Ub-probe-F	5’-ACGGAATTCCATATGTGGAGCCACCCGCAGTTCGAAAAAGGCAGC ATGCAGATCTTCGTGAAGACCC -3’
Ub-probe-R	5’- GCGATTTGCTCTTCCGCATCCGCGCAGGCGCAGTACCAGGTGCAGTGTACT -3’
NEDD8-probe-F	5’- ACGGAATTCCATATGTGGAGCCACCCGCAGTTCGA -3’
NEDD8-probe-R	5’- GCGATTTGCTCTTCCGCAACCGCGCAGGGCCAGAACCA -3’

The underlined sequences are the restriction sites, Sal I in primer U-F- Sal I, Hind III in U480-R-Hind III, and U323-R-Hind III, Pst I in GST-R-Pst I, Nde I in Ub-probe-F and NEDD8-probe-F, Sap I in Ub-probe-R and NEDD8-probe-R, respectively. The boldfaced lowercase sequences are the mutation sites.

## Supplementary Materials

Supplementary materials can be found at https://www.mdpi.com/1422-0067/21/5/1783/s1. Table S1. Optimization of wild type UL36(480) gene of MDV; Table S2. Accession numbers of UL36 homologs encoded by different herpesvirus species; Table S3. Accession numbers of UL36 encoded by various Gallid herpesvirus 2 strains; Figure S1. Characterization of hydrolysis activity of UL36(480)^WT^ on rhodamine-conjugated Ub substrates.

## Abbreviations

CCHFV	Crimean–Congo hemorrhagic fever virus
CPIC	Complete protease inhibitor cocktail
DUB	Deubiquitinase
DUGV	Dugbe virus
EAV	Equine arteritis virus
EBV	Epstein–Barr virus
ECL	Enhanced chemiluminescence
GaHV-2	Gallid alphaherpesvirus 2
HCMV	Human cytomegalovirus
HSV	Herpes simplex virus
KSHV	Kaposi’s sarcoma-associated herpesvirus
MD	Marek’s disease
MDV	Marek’s disease virus
MERS-CoV	Middle East respiratory syndrome-related coronavirus
MESNa	Mercaptosulfonate sodium salt
MJD	Machado–Josephin domain protease
OTU	Ovarian tumor proteases
PMSF	Phenylmethanesulfonyl fluoride
PRRSV	Porcine reproductive and respiratory syndrome virus
PRV	Pseudorabies virus
SARS-CoV	Severe acute respiratory syndrome-coronavirus
Ub	Ubiquitin
Ub-AMC	Ubiquitin 7-amido-4-methylcoumarin
UbL	Ubiquitin-like modifier
UCH	Ubiquitin C-terminal hydrolase
USP	Ubiquitin-specific protease
