# Supplementary material for: UL36 Encoded by Marek’s Disease Virus Exhibits Linkage-Specific Deubiquitinase Activity
Source: Int J Mol Sci. 2020 Mar 5;21(5):1783. doi: 10.3390/ijms21051783 (PMC7084888; doi:10.3390/ijms21051783)
Supplement: Supplementary file 1 [file ijms-21-01783-s001.zip › Supplementary data legends.docx]

**Supplementary data legends of ‘UL36 encoded by Marek’s Disease Virus exhibits linkage-specific deubiquitinase activity’**

**Table S1. Optimization of wild type *UL36(480)* gene of MDV. Original Sequence,** the original gene sequence of wild type *UL36(480)* encoded by virulent MDV strain J-1 genome (GeneBank ID KU744555); **Optimized Sequence,** the optimized sequence based on ‘**Original sequence**’ using software Codon OptimWiz.

**Table S2.** Accession numbers of UL36 homologs encoded by different herpesvirus species.

**Table S3.** Accession numbers of UL36 encoded by various Gallid herpesvirus 2 strains.

**Figure S1.** Characterization of hydrolysis activity of UL36(480)WT on rhodamine-conjugated Ub substrates. The fluorescence traces (red line) of rhodamine released on the hydrolysis of rhodamine-conjugated Ub substrates by wild type UL36(480), UL36(480)WT. Control (grey line), the group contained an equal volume of buffer but no UL36(480)WT.
