# Supplementary figures and images for: UL36 Encoded by Marek’s Disease Virus Exhibits Linkage-Specific Deubiquitinase Activity
Source: Int J Mol Sci. 2020 Mar 5;21(5):1783. doi: 10.3390/ijms21051783 (PMC7084888; doi:10.3390/ijms21051783)

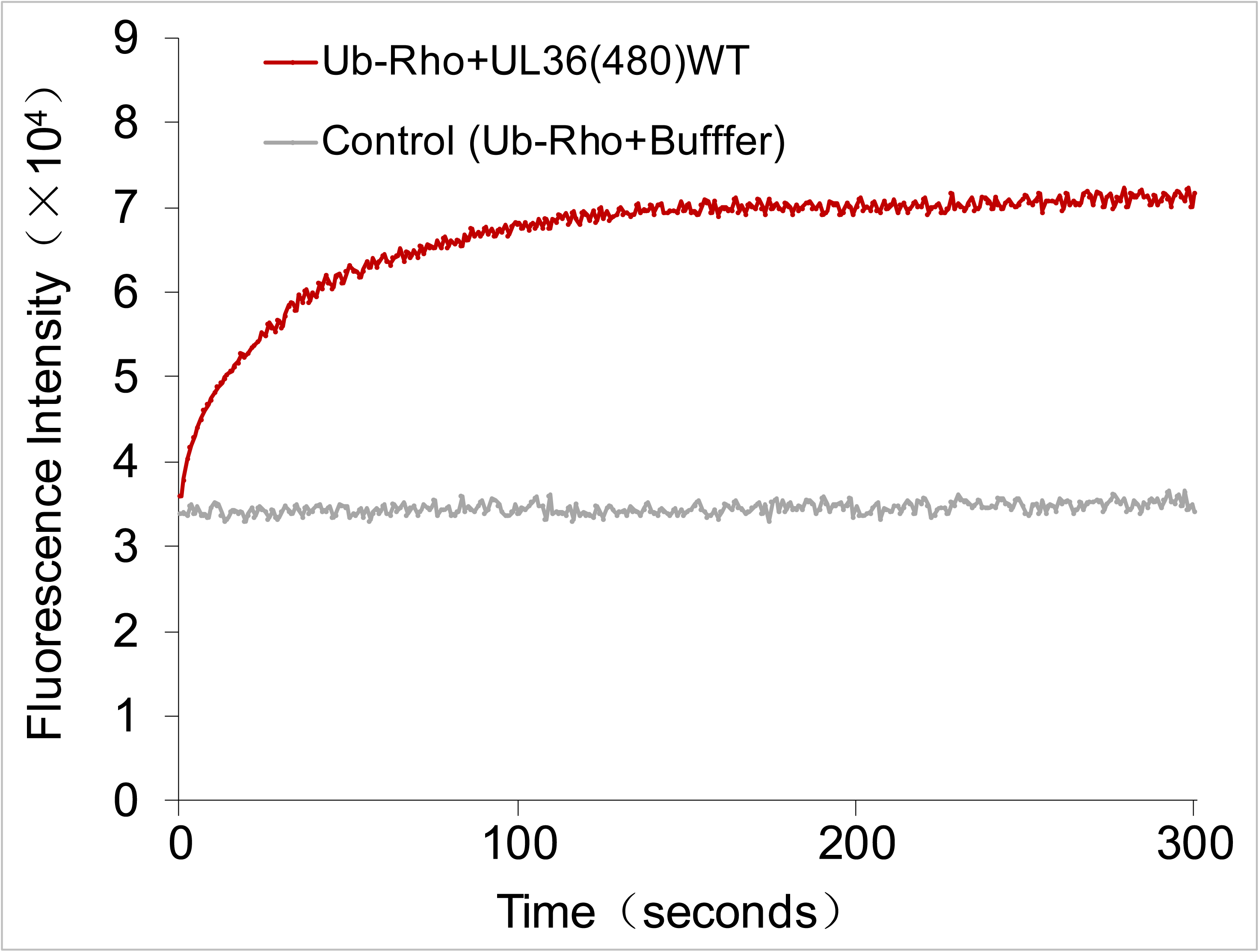

Supplement: Supplementary file 1 [file ijms-21-01783-s001.zip › Figure S1.tif]
